# Supplementary material for: ATAD5 promotes replication restart by regulating RAD51 and PCNA in response to replication stress
Source: Nat Commun. 2019 Dec 16;10:5718. doi: 10.1038/s41467-019-13667-4 (PMC6914801; doi:10.1038/s41467-019-13667-4)
Supplement: Supplementary file 1 — Supplementary Information [file 41467_2019_13667_MOESM1_ESM.pdf]

Supplementary Information

ATAD5 promotes replication restart by regulating RAD51 and PCNA in response to replication stress

Park et al.

Supplementary Figures

Supplementary Figure 1

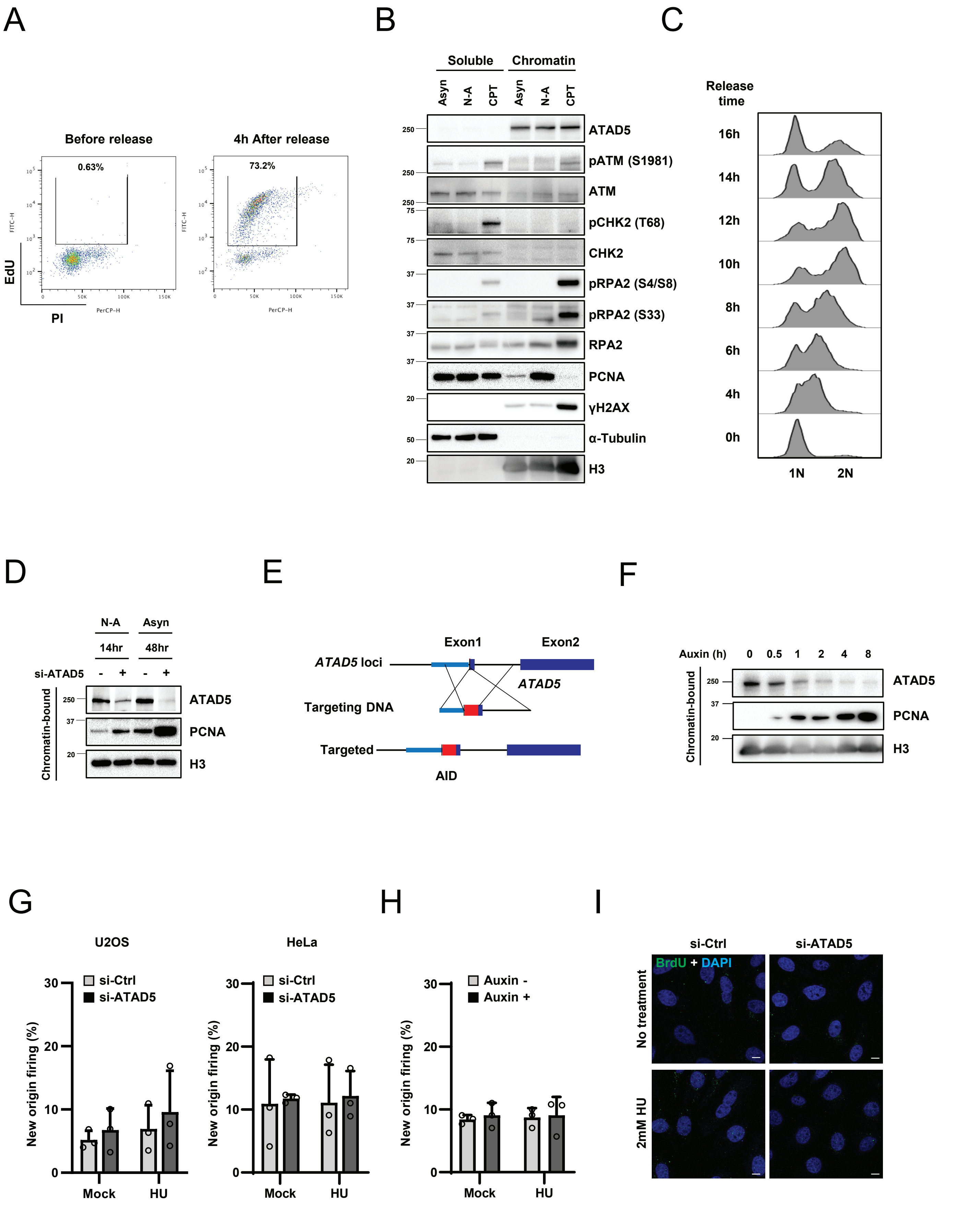

Supplementary Figure 1. ATAD5 promotes replication fork restart at stalled replication forks.

(A–C) U2OS cells arrested at the G1/S boundary (the Noco-APH condition) were released from growth arrest in normal media for the indicated time before being collected for cell cycle analysis or immunoblotting. (A) Cells were pulse-labeled with EdU for 30 min before collection. DNA was stained with propidium iodide. (B) Soluble and chromatin-bound protein fractions were isolated from cells in asynchronous (Asyn) or the Noco-APH (N-A) condition and subjected for immunoblotting. Cells treated with 1 μM camptothecin (CPT) for 4 h were used as a control. (C) The cellular DNA content was measured by propidium iodide staining and flow cytometry. (D) Chromatin-bound proteins were isolated from U2OS cells in asynchronous (Asyn) or the Noco-APH (N-A) condition and subjected for immunoblotting. (E) A schematic diagram showing the generation of a U2OS cell line expressing an endogenously auxin-inducible degron (AID) tagged ATAD5 (U2OS-ATAD5^{AID}). The *AID* gene was inserted between the ATG start codon and the second codon of the endogenous *ATAD5* gene. (F) U2OS-ATAD5^{AID} cells were treated for the indicated times with auxin and chromatin-bound proteins were separated by SDS PAGE and subjected for immunoblotting with indicated antibodies. (G, H) The percentages of newly fired origins were calculated from data in Figures 1D and 1E and displayed. Error bars represent standard deviation of the mean (n = 3). (I) HeLa cells transfected with *ATAD5* siRNA under the Noco-APH condition were labeled with BrdU for 10 min, washed, and incubated in culture media containing 2 mM HU for 4 h before fixation. Single-stranded DNA with exposed BrdU was visualized by staining with an anti-BrdU antibody under the native non-denaturing condition. Scale bar: 20 μm.

Supplementary Figure 2

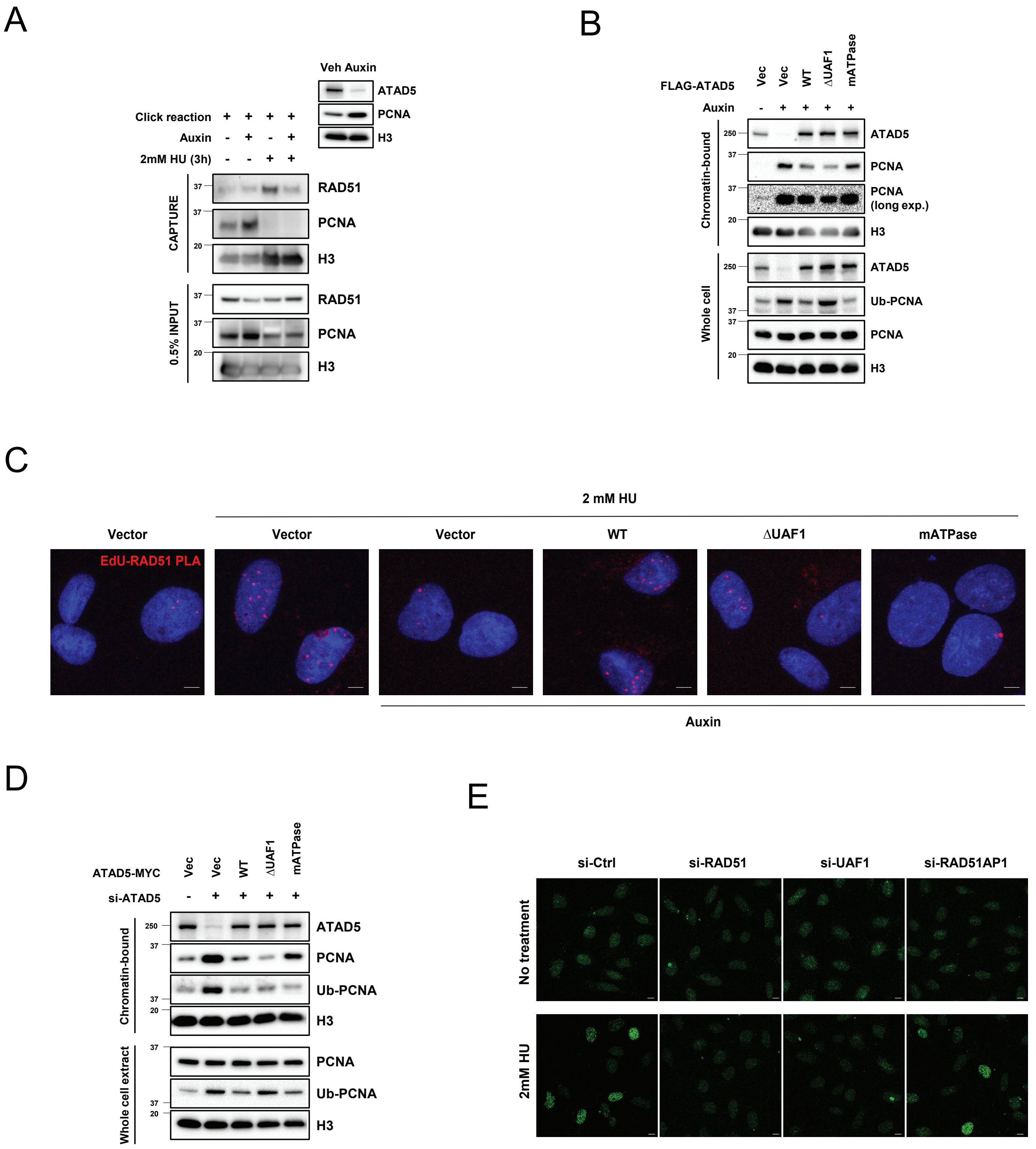

Supplementary Figure 2. ATAD5 promotes RAD51 recruitment to stalled replication forks, which depends on the PCNA unloading activity of ATAD5.

(A) U2OS cells expressing ATAD5^{AID} were pre-treated with auxin. Then, cells were processed for the isolation of proteins on nascent DNA (iPOND) assay and immunoblotting. The right panel shows chromatin-bound proteins extracted from a portion of cells by immunoblotting. (B) U2OS cells expressing ATAD5^{AID} were transfected with a cDNA expression vector, treated with auxin, and chromatin bound fractions were prepared for immunoblotting. (C) Representative SIRF images of Figure 2E. Scale bar: 5 μm. (D) U2OS-TetOn-ATAD5 cell lines expressing wild type (WT) ATAD5 or two ATAD5 mutants (∆UAF1, UAF1 interaction defective mutant; mATPase, ATPase domain mutant) in a doxycycline (Dox)-inducible manner were established. After Dox treatment, cells were transfected with *ATAD5* siRNA. After 48 h, chromatin-bound proteins or whole cell proteins were extracted and subjected to immunoblotting with indicated antibodies. (E) Representative native BrdU staining images of Figure 2M. Scale bar: 20 μm.

Supplementary Figure 3

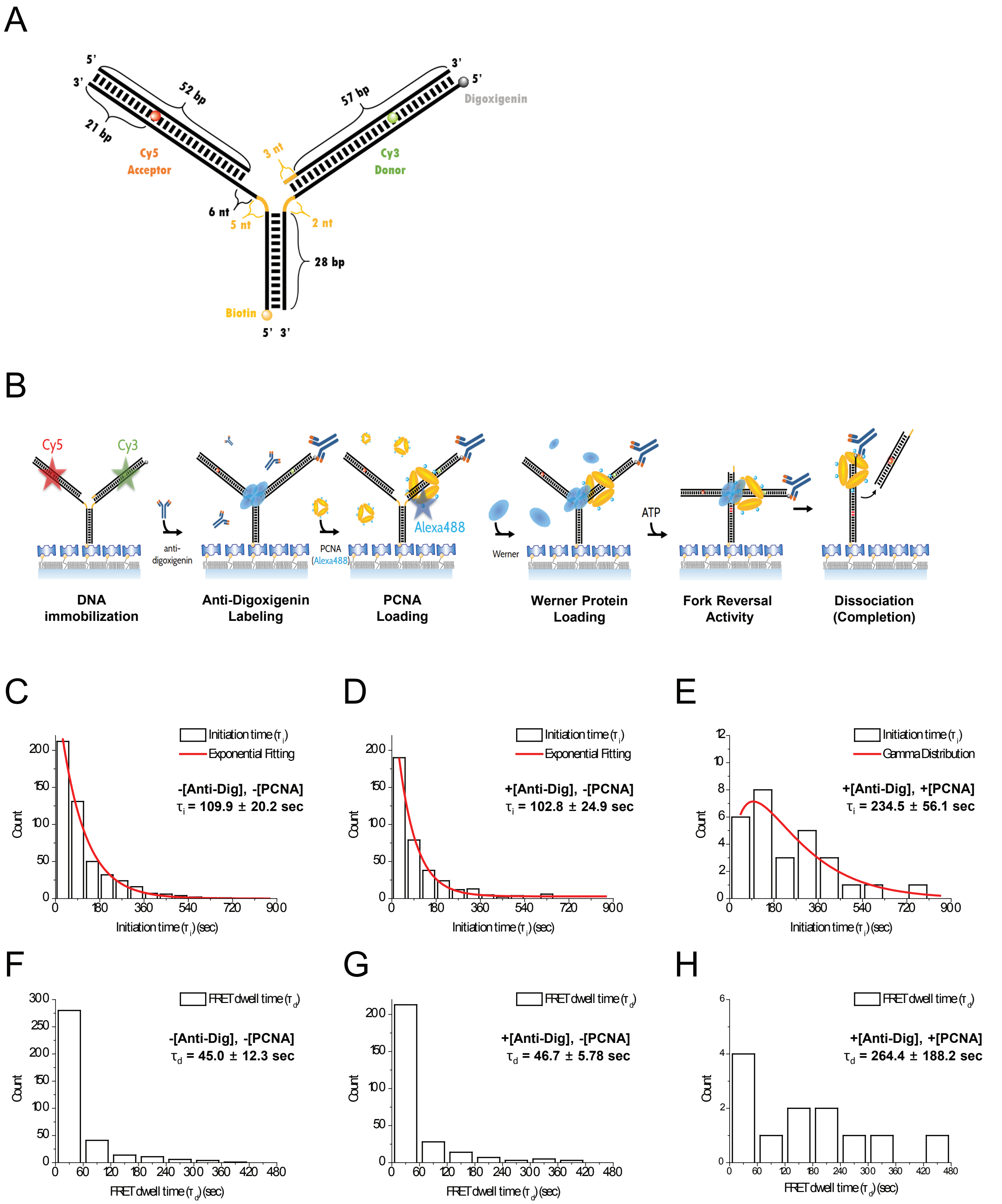

Supplementary Figure 3. A single-molecule FRET experiment.

(A) The design of a model replication fork. (B) A single-molecule FRET experimental scheme to observe fork reversal activity of WRN. Model replication forks were immobilized on Quartz slide. After blocking the free end of the lagging strand with anti-digoxigenin, PCNAs and WRN were subsequently loaded on the replication fork. Between each step, all free proteins were removed from the reaction chamber. The fork reversal reaction of WRN was activated by injecting image buffer containing ATP-Mg^{2+}. The formation of the four-way junction, and dissociation of daughter duplex upon the completion of fork reversal activity were monitored using a total-internal-reflection fluorescence microscope. (C-E) Distributions of the initiation time of fork reversal activity in three different reaction conditions: i) neither anti-digoxigenin nor PCNA was loaded (C), only anti-digoxigenin was loaded (D), both anti-digoxigenin and PCNA were loaded (E). (F-H) Distributions of FRET dwell time of fork reversal activity in three different reaction conditions: i) neither anti-digoxigenin nor PCNA was loaded (F), only anti-digoxigenin was loaded (G), both anti-digoxigenin and PCNA were loaded (H).

Supplementary Figure 4

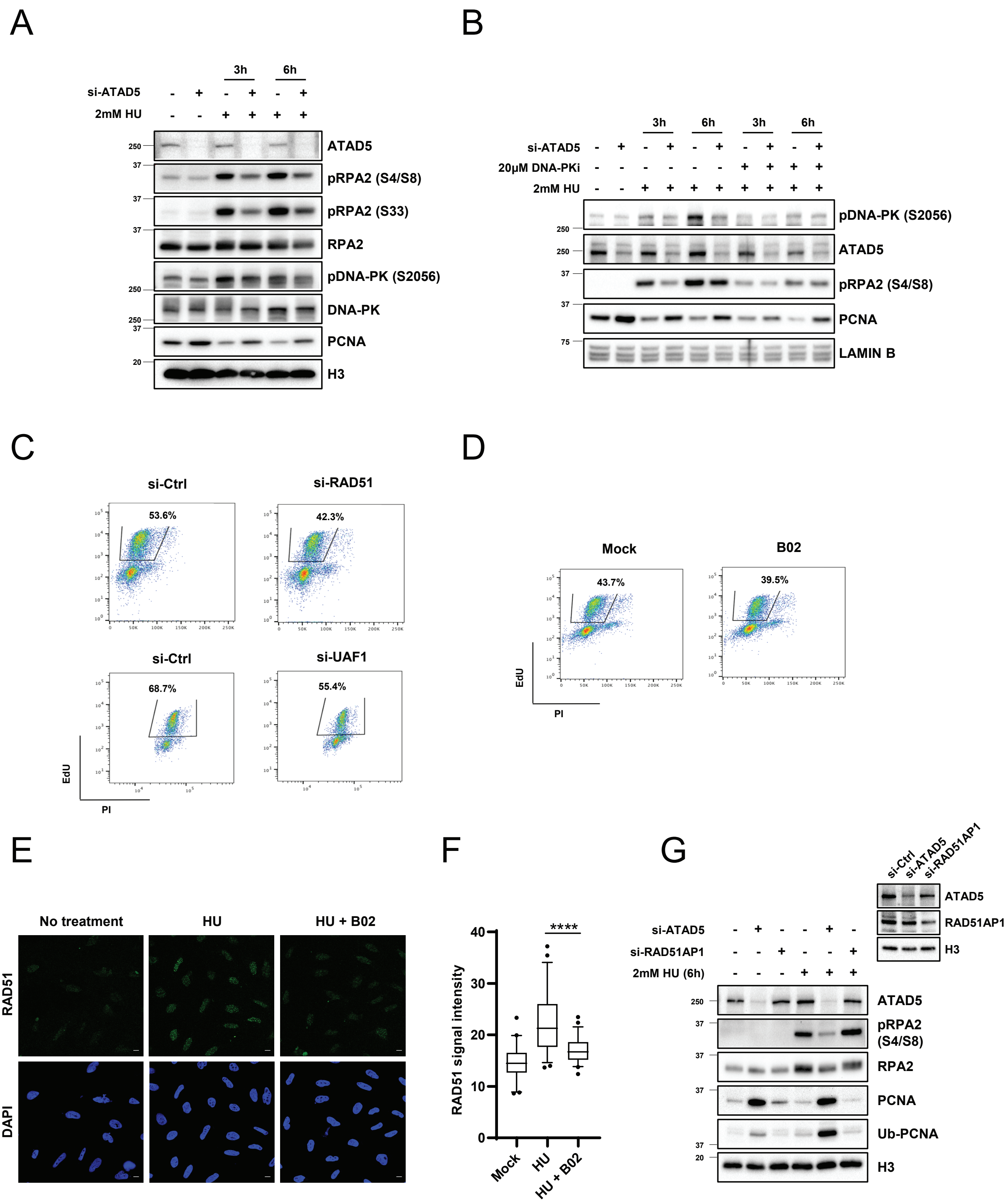

Supplementary Figure 4. ATAD5 promotes generation of single-stranded DNA-associated breaks in response to replication stress.

(A) HeLa cells transfected with *ATAD5* siRNA under the Noco-APH condition were treated with 2 mM HU for 3 or 6 h. Chromatin-bound proteins were fractionated and subjected for immunoblotting. (B) U2OS cells transfected with *ATAD5* siRNA under the Noco-APH condition were treated with 2 mM HU or 20 μM DNA-PK inhibitor (DNA-PKi, Nu7026) for the indicated times. Chromatin-bound proteins were fractionated and subjected for immunoblotting. (C) U2OS cells transfected with siRNAs under the Noco-APH condition were released from growth arrest in normal media for 4 h before being collected for cell cycle analysis. Cells were pulse-labeled with EdU for 30 min before collection. (D) U2OS cells treated with a RAD51 inhibitor (B02) at the time of release from aphidicolin treatment for 4 h before being collected for cell cycle analysis. (E, F) U2OS cells treated with a RAD51 inhibitor (B02) at the time of release from aphidicolin for 4 h were treated with 2 mM HU for 3 h before fixation. Cells were then subjected for immunostaining with an anti-RAD51 antibody. (E) Representative images of chromatin-bound RAD51. Scale bar: 20 μm. (F) The intensity of chromatin-bound RAD51 staining was quantified and plotted. Statistical analysis: *t*-test; **** $p < 0.0001$. (G) U2OS cells transfected with *RAD51AP1* siRNA under the Noco-APH condition were treated with 2 mM HU for 6 h. Chromatin-bound proteins were separated by SDS PAGE and subjected for immunoblotting with indicated antibodies. The right panel shows whole cell proteins extracted from a portion of cells in (G), separated by SDS-PAGE and immunoblotted.

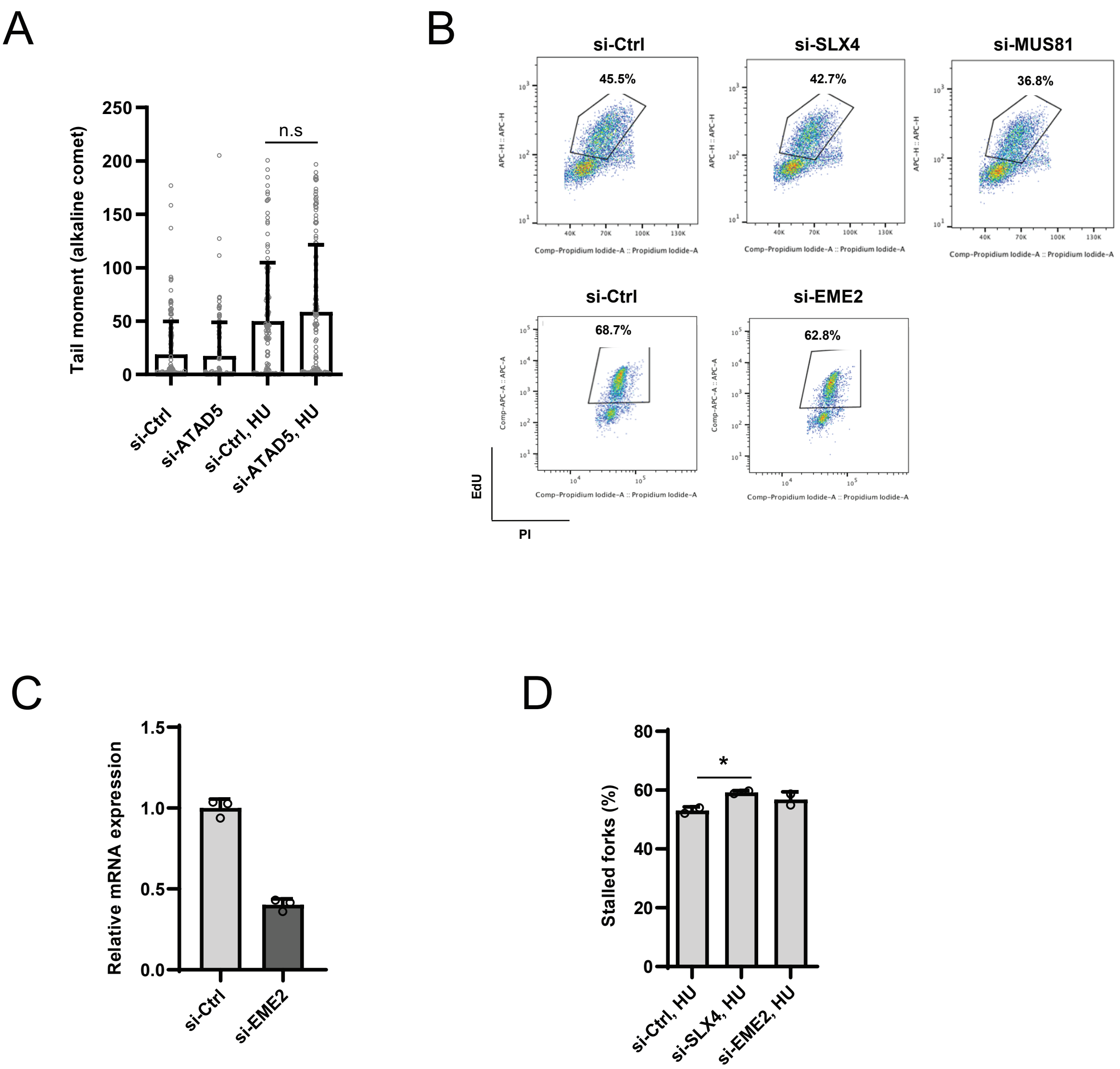
A
Tail moment (alkaline comet)
250
200
150
100
50
0
n.s
si-Ctrl
si-ATAD5
si-Ctrl, HU
si-ATAD5, HU
B
si-Ctrl
45.5%
si-SLX4
42.7%
si-MUS81
36.8%
si-Ctrl
68.7%
si-EME2
62.8%
EdU
PI
C
Relative mRNA expression
1.5
1.0
0.5
0.0
si-Ctrl
si-EME2
D
Stalled forks (%)
80
60
40
20
0
*
si-Ctrl, HU
si-SLX4, HU
si-EME2, HU

Supplementary Figure 5. ATAD5 promotes generation of MUS81-mediated single-stranded DNA-associated breaks in response to replication stress.

(A) U2OS cells transfected with *ATAD5* siRNA under the Noco-APH condition were treated with 2 mM HU for 6 h before being collected for an alkaline COMET assay. The tail moment was calculated from ~150 cells and plotted. Three independent experiments were performed, and one representative result is displayed. Error bars represent standard deviation of the mean (n = 3). Statistical analysis: two-tailed Student's *t*-test; n.s. not significant. (B) U2OS cells transfected with siRNAs under the Noco-APH condition were released from growth arrest in normal media for 4 h before being collected for cell cycle analysis. Cells were pulse-labeled with EdU for 30 min before collection. (C) Total RNA was extracted from a portion of cells transfected with *EME2* siRNA in Figure 5F and then *EME2* mRNA level was measured with the quantitative PCR (n = 3). (D) U2OS cells transfected with siRNAs under the Noco-APH condition were pre-labeled with Cl-dU for 20 min and then treated with 2 mM HU for 2 h. Cells were then washed and labeled with I-dU for 30 min before being collected for a DNA combing assay (n = 3). The percentages of restarted and newly fired forks are displayed. (C, D) Error bars represent standard deviation of the mean. Three independent experiments were performed. Statistical analysis: *t*-test; * $p < 0.05$.

Supplementary Tables

Supplementary Table 1. Putative ATR phosphorylation sites tested

Protein	Site tested	Reference
ATAD5	S621	Matsuoka et al., 2007[1]
ATAD5	T627	Dephoure et al., 2008[2]
ATAD5	S688	Stokes et al., 2007[3]
ATAD5	S817	Sharma et al., 2014[4]
UAF1	S611	Sharma et al., 2014[4]
RAD51	T134	Flott et al., 2011[5]
RAD51	T309	Sorensen et al., 2005[6]

Supplementary Table 2. Oligonucleotides for small interfering RNA (siRNA) and single guide RNA (sgRNA)

Control siRNA	Bioneer	Cat#SN-1002
ATAD5 siRNA (GUAUAUUUCUCGAUGUACA)	Lee et al., 2013[7]	N/A
RAD51 siRNA	Dharmacon	Cat# L-003530-00
SLX4 siRNA	Dharmacon	Cat# L-014895-00-0010
MUS81 siRNA	Dharmacon	Cat# L-016143-00-0010
EME2 siRNA (GAGAGCCAGUGGCAAGAGA)	Bioneer	N/A
UAF1 siRNA	Dharmacon	Cat# L-016462-01
RAD51AP1 (CCUCAUAUCUCUAAUUGCA)	Bioneer	N/A
USP1 siRNA (AGCUACAAGUGAUACAUUA)	Bioneer	N/A
RFC4 siRNA	Bioneer	Cat#1128443V
sgRNA set 1 (CCGTCTCCTAGGACCTCACGCGG)	Marcrogen	N/A
sgRNA set 2 (CGGGAAGCGGGGAGTATGGTGGG)	Marcrogen	N/A

Supplementary Table 3. Oligonucleotides for Single molecule FRET

Leading (Daughter)	TCG ACA GGT CAT GGC CGT ACA T*GA TAT CCT CGA GCG GTC CTG TTG CAA CTT A
Lagging (Daughter)	TCA GAG TGT TAA GTT GCA ACA GGA CCG CTC GAG GAT* ATC ATG TAC GGC CAT GAC CTG TCG
Leading (Parent)	Biotin-TGT TAA CCC TAA CCC TAA GAA TTC GGC TTA AGT GAG TGT TAA GTT GCA ACA GGA CCG CTC GAG GAT ATC ATG TAC GGC CAT GAC CTG TCG A
Lagging (Parent)	Amino-CGA CAG GTC ATG GCC GTA CAT GAT ATC CTC GAG CGG TCC TGT TGC AAC TTA ACA CTC TGA ATA GCC GAA TTC TTA GGG TTA GGG TTA ACA ATA GCC GAA T

Supplementary References

1. Matsuoka S, *et al.* ATM and ATR substrate analysis reveals extensive protein networks responsive to DNA damage. *Science* **316**, 1160-1166 (2007).

2. Dephoure N, *et al.* A quantitative atlas of mitotic phosphorylation. *Proc Natl Acad Sci U S A* **105**, 10762-10767 (2008).

3. Stokes MP, *et al.* Profiling of UV-induced ATM/ATR signaling pathways. *Proc Natl Acad Sci U S A* **104**, 19855-19860 (2007).

4. Sharma K, *et al.* Ultradeep human phosphoproteome reveals a distinct regulatory nature of Tyr and Ser/Thr-based signaling. *Cell Rep* **8**, 1583-1594 (2014).

5. Flott S, Kwon Y, Pigli YZ, Rice PA, Sung P, Jackson SP. Regulation of Rad51 function by phosphorylation. *EMBO Rep* **12**, 833-839 (2011).

6. Sorensen CS, *et al.* The cell-cycle checkpoint kinase Chk1 is required for mammalian homologous recombination repair. *Nat Cell Biol* **7**, 195-201 (2005).

7. Lee KY, Fu H, Aladjem MI, Myung K. ATAD5 regulates the lifespan of DNA replication factories by modulating PCNA level on the chromatin. *J Cell Biol* **200**, 31-44 (2013).
